# Assessment of Vitamin D Supplementation on Articular Cartilage Morphology in a Young Healthy Sedentary Rat Model

**DOI:** 10.3390/nu11061260

**Published:** 2019-06-03

**Authors:** Marta Anna Szychlinska, Rosa Imbesi, Paola Castrogiovanni, Claudia Guglielmino, Silvia Ravalli, Michelino Di Rosa, Giuseppe Musumeci

**Affiliations:** 1 Department of Biomedical and Biotechnological Sciences, Human Anatomy and Histology Section, School of Medicine, University of Catania, 95123 Catania, Italy; marta.sz@hotmail.it (M.A.S.); roimbesi@unict.it (R.I.); pacastro@unict.it (P.C.); claudiaguglielmino11@gmail.com (C.G.); silviaravalli@gmail.com (S.R.); mdirosa@unict.it (M.D.R.); 2 Research Center on Motor Activities (CRAM), University of Catania, 95123 Catania, Italy

**Keywords:** vitamin D, diet, functional food, articular cartilage, morphology, immunohistochemistry

## Abstract

Deficiency in vitamin D (Vit D) has been widely associated with several musculoskeletal diseases. However, the effects of the exogenous Vit D supplementation are still unclear in the prevention of the latter, especially in the cartilage developmental period. The aim of this study was to compare the effects of Vit D supplementation and restriction on the articular cartilage development in healthy young sedentary rats. To this aim, twelve nine-week-old healthy Sprague–Dawley male rats were subjected to Vit D-based experimental diets: R, with a content in Vit D of 1400 IU/kg; R-DS, with a Vit D supplementation (4000 IU/kg); R-DR, with a Vit D restriction (0 IU/kg) for 10 weeks. The morphology, thickness and expression of cartilage-associated molecules such as collagen type II/X, lubricin and Vit D receptor (VDR), were assessed. Histological, histomorphometric and immunohistochemical evaluations were made on rat tibial cartilage samples. In the present experimental model, restriction of Vit D intake induced: The lower thickness of cartilage compared both to R (*p* = < 0.0001) and R-DS (*p* = < 0.0001); reduction of proteoglycans in the extracellular matrix (ECM) compared both to R (*p* = 0.0359) and R-DS (*p* = < 0.0001); decreased collagen II (Col II) with respect both to R (*p* = 0.0076) and R-DS (*p* = 0.0016); increased collagen X (Col X) immunoexpression when compared both to R (*p* = < 0.0001) and R-DS (*p* = < 0.0001), confirming data from the literature. Instead, supplementation of Vit D intake induced: Higher cartilage thickness with respect both to R (*p* = 0.0071) and R-DR (*p* = < 0.0001); increase of ECM proteoglycan deposition compared both to R (*p* = 0.0175) and R-DR (*p* = < 0.0001); higher immunoexpression of lubricin with respect both to R (*p* = 0.001) and R-DR (*p* = 0.0008). These results suggest that Vit D supplementation with diet, already after 10 weeks, has a favorable impact on the articular cartilage thickness development, joint lubrication and ECM fibers deposition in a young healthy rat model.

## 1. Introduction

The importance of vitamin D (Vit D) in the musculoskeletal system development and function, is well known, as reported in several studies testifying a range of effects of Vit D deficiency on cartilage, bone and muscle tissues [1,2,3].

Articular cartilage is an avascular tissue characterized by chondrocytes surrounded by an ECM, rich in water and consisting of collagen type II and proteoglycans with sulfated glycosaminoglycan (GAG) side chains. When an imbalance between catabolism and anabolism occurs in the articular cartilage, joint impairment may manifest as a typical condition of some diseases such as osteoarthritis (OA), characterized by cartilage degeneration and progressive loss [4,5].

Healthy cartilage turnover depends on suitable accessibility of Vit D [1]. Suboptimal levels may lead to OA via its adverse effects on cartilage metabolism [6,7]. Indeed, it has been demonstrated that adequate levels of Vit D stimulate mature chondrocytes to synthesize proteoglycan matrix proteins [8,9]. On the other hand, its low levels increase MMP activity [10]. Therefore, low levels of exogenous Vit D may alter the stability of cartilage metabolism by reducing the synthesis of proteoglycan and/or increasing the MMPs activity, leading to cartilage loss. Data from the literature show that Vit D deficiency is responsible for proteoglycans and matrix loss in the animal model [11] and it is also associated with progression of OA, in humans [6,7,12]. Scientific data support the beneficial effects of Vit D as an immunomodulatory and anti-inflammatory agent in OA [7,13], even if the reported data are controversial [14,15]. It has been shown that Vit D influences the state of the joints through the action of VDR, whose polymorphism has been associated with OA [16,17]. Deficiency in Vit D has been associated with several other musculoskeletal diseases, such as osteomalacia, osteopenia, osteoporosis, increased risk of fracture and muscle weakness [18]. However, the exogenous Vit D supplementation with diet has been quite poorly studied and it is still unclear whether it has beneficial effects in the prevention of Vit D-deficiency related diseases of musculoskeletal tissues, especially during the developmental period.

From the mentioned above scientific data, it is evident how complex is the relation between the musculoskeletal system and molecular action of Vit D. For this reason, the aim of the present study was to evaluate the effects of exogenous Vit D supplementation and restriction on morphology and expression of some chondrocyte markers such as lubricin, Col II and hypertrophic marker collagen type X (Col X), in tibial articular cartilage of young healthy sedentary rats. The hypothesis of the present study was that Vit D supplementation may support the development of the articular cartilage morphology during its developmental period by determining the increase in its thickness, which may better support the mechanical loading and permit in this way to perform the native function of articular cartilage.

## 2. Materials and Methods

### 2.1. Breeding and Housing of Animals

Twelve nine-week-old healthy Sprague–Dawley male rats (Envigo RMS S.r.l., Udine, Italy), with an average body weight of 271 ± 25 g, were housed in polycarbonate cages (cage dimensions: 10.25″ W × 18.75″ D × 8″ H) at controlled temperature (20–23 °C) and humidity. Only male rats were used to avoid different results related to different genders. During the whole period of the research, with free access to water and food and a photoperiod of 12 h light/dark at the “Center for Advanced Preclinical In Vivo Research (CAPIR)”. Rats were allowed to adopt one week to their environment before the experiments began. At the end of the experimental period (10 weeks), the animals were humanely sacrificed by exposure to a chamber filled with carbon dioxide until one minute after breathing stopped and then were decapitated. After euthanasia, articular cartilage samples from tibia were used to perform the histological, histochemical, histomorphometric and immunohistochemical evaluation. In order to maximize the information obtained from the minimum resource and to generate statistically robust data, the power analysis sample size was performed using the G*Power3.1 calculator software (Düsseldorf University, Düsseldorf, Germany) [19]. All of the experiments were designed to minimize animal suffering and to use the minimum number of animals required to achieve a valid statistical evaluation according to the principles of the 3R’s (replacement, reduction and refinement). All of the procedures conformed to the guidelines of the Institutional Animal Care and Use Committee (I.A.C.U.C.) at the Center for Advanced Preclinical In Vivo Research (CAPIR), University of Catania, and approved by Italian Ministry of Health, Protocol n. 2112015-PR of the 14.01.2015 (14 January 2015). The experiments were conducted in accordance with the European Community Council Directive (86/609/EEC) and the Italian Animal Protection Law (116/1992).

### 2.2. Experimental Design

Three different diets were used for the experiment, provided by Mucedola s.r.l. (Settimo Milanese, Milan, Italy) by adding different contents of cholecalciferol (Vit D₃) in a standard chow. In particular, a regular diet with a content in Vit D₃ of 1400 IU/kg (R), a regular diet with Vit D₃ supplementation (4000 IU/kg) (R-DS) and a regular diet with Vit D₃ restriction (0 IU/kg) (R-DR). The composition of the experimental diets is reported in Table 1. The 12 animals were divided into three groups: R, control rats, fed with R diet (*n* = 4); R-DS, rats fed with R-DS diet (*n* = 4); R-DR, rats fed with R-DR diet (*n* = 4). The experimental protocol was carried out for a period of 10 weeks and body weights, food and drink consumptions were monitored three days per week throughout the experiment. At the end of the experiment, rats were sacrificed, as above reported, in order to obtain cartilage tissue in which evaluations were performed.

### 2.3. Histology

The tibias explantation procedure and the subsequent cleaning of soft tissues were performed as previously described [20]. Specimens were incubated in a decalcifying solution (14% EDTA, PH: 7,2) for 7–10 days, washed for one h and then were fixed in 10% neutral buffered formalin (Bio-Optica, Milan, Italy). After overnight washing, they were embedded in paraffin as previously described [21]. The samples were placed in the cassettes in longitudinal and cross directions after wax infiltration. Tissue samples (4–5 μm) were cut from paraffin blocks by a rotary manual microtome (Leica RM2235, Milan, Italy) and then mounted on silane-coated slides (Menzel-Gläser, Braunschweig, Germany) and preserved at room temperature. Afterwards, the sections were dewaxed in xylene, hydrated by graded ethanol, and stained by Hematoxylin and Eosin (H&E), Alcian Blue and Mallory’s trichrome stainings for detection of structural alterations, histochemical evaluation and histomorphometric measurements (thickness of the articular cartilage). The slides were examined with a Zeiss Axioplan light microscope (Carl Zeiss, Oberkochen, Germany), and pictures were taken with a digital camera (AxioCam MRc5, Carl Zeiss, Oberkochen, Germany).

### 2.4. Histomorphometric Analysis

Four fields, the area of which was about 550,000 µm^2^, randomly selected from sections (four from each rat), were analysed for morphometric analysis. The thickness of the articular cartilage was considered and calculated using software for image acquisition (AxioVision Release 4.8.2-SP2 Software, Carl Zeiss Microscopy GmbH, Jena, Germany). The data were expressed as mean ± standard deviation (SD). The statistical significance of the results was then evaluated. Digital micrographs were taken using the Zeiss Axioplan light microscope (Carl Zeiss, Oberkochen, Germany), using a lens with a magnification of ×10, i.e., total magnification 100) fitted with a digital camera (AxioCam MRc5, Carl Zeiss, Oberkochen, Germany). Three blinded investigators (two anatomical morphologists and one histologist) made the evaluations that were assumed to be correct if the recorded values had no statistically significant difference. In case of a dispute concerning interpretation, the case was reconsidered to reach a unanimous agreement.

### 2.5. Immunohistochemistry (IHC)

Articular cartilage samples were processed for immunohistochemical analysis as previously described [22]. In detail, the slides were dewaxed in xylene, hydrated by graded ethanol, incubated for 30 min in 0.3% hydroperoxyl (HO_2_)/methanol to remove endogenous peroxidase activity and then rinsed in phosphate-buffered saline (PBS; Bio-Optica, Milan, Italy) for 20 min. In order to unmask the antigenic sites, the samples were stored in capped polypropylene slide holders with citrate buffer (10 mM citric acid, 0.05% Tween 20, pH 6.0; Bio-Optica, Milan, Italy) and heated for 5 min for three times through a microwave oven (750 W, LG Electronics Italia S.p.A., Milan, Italy). In order to prevent non-specific binding of the antibodies, a blocking step with 5% bovine serum albumin (BSA, Sigma, Milan, Italy) in PBS for one h in a moist chamber was performed before the application of the primary antibodies. The sections were then incubated overnight at 4 °C with the following antibodies: Rabbit polyclonal Anti-Col II antibody (ab34712; Abcam, Cambridge, UK), work dilution in PBS (Bio-Optica, Milan, Italy) 10 μg/mL; rabbit polyclonal Anti-Col X antibody (ab58632; Abcam, Cambridge, UK), work dilution in PBS (Bio-Optica, Milan, Italy) 10 μg/mL; rabbit polyclonal anti-lubricin antibody (ab28484; Abcam, Cambridge, UK), work dilution in PBS (Bio-Optica, Milan, Italy) 4 μg/mL; rat monoclonal anti-VDR (ab115495; Abcam, Cambridge, UK), work dilution in PBS (Bio-Optica, Milan, Italy) 10 μg/mL. The samples were then coated with a biotinylated antibody (horseradish peroxidase (HRP)-conjugated anti-goat and anti-rabbit were used as secondary antibodies), and the immune complexes were detected with peroxidase-labeled streptavidin (labelled streptavidin-biotin (LSAB) + System-HRP, K0690, Dako, Glostrup, Denmark), after incubation for 10 min at room temperature. The immunoreaction was detected by incubating the sections for 2 min in a 0.1% 3,3′-diaminobenzidine, 0.02% hydrogen peroxide solution (DAB substrate Chromogen System; Dako, Denmark). The slides were lightly counterstained with Mayer’s Hematoxylin (Histolab Products AB, Goteborg, Sweden) and mounted in GVA mount (Zymed, Laboratories Inc., San Francisco, CA, USA).

### 2.6. Computerised Densitometric Measurements and Image Analysis

Four fields, the area of which was about 550,000 µm^2^, randomly selected from each section, were analysed for histochemical evaluation of Alcian Blue staining which detects mucosubstance content (GAGs) and Mallory’s trichrome staining which detects the fibrous component (collagen fibres). It was used as an image analysis software (AxioVision Release 4.8.2-SP2 Software, Carl Zeiss Microscopy GmbH, Jena, Germany), which quantifies the level of staining in the densitometric count (pixel^2^)/μm^2^ of articular cartilage.

To quantify the level of immunostaining of positive anti-Col II, anti-Col X, anti-lubricin and anti-VDR antibodies immunolabelling, the same software was used for calculating the densitometric count (pixel^2^)/μm^2^ of the immunostained articular cartilage area in four fields, the area of which was about 550.000 µm^2^, randomly selected from slides. Digital micrographs were taken using the Zeiss Axioplan light microscope (Carl Zeiss, Oberkochen, Germany), using a lens with a magnification of ×10, i.e., total magnification 100) fitted with a digital camera (AxioCam MRc5, Carl Zeiss, Oberkochen, Germany). Three blinded investigators (two anatomical morphologists and one histologist) made the evaluations that were assumed to be correct if the recorded values had no statistically significant difference. If disputes concerning interpretation occurred, a unanimous agreement was reached after sample re-evaluation [23].

### 2.7. Statistical Analysis

The statistical analysis was performed using GraphPad Instat^®^ Biostatistics version 3.0 software (GraphPad Software, Inc., La Jolla, CA, USA) [21,22]. Datasets were tested for normal distribution with the Kolmogorov–Smirnov test. All variables were normally distributed. Two-way-ANOVA (Tukey’s multiple comparisons test) was used for comparisons between more than two groups. *p*-values of less than 0.05 were considered statistically significant; more than 0.05, they were considered not significant (ns). The data are presented as the mean ± SD.

## 3. Results

### 3.1. Histology and Histomorphometric Analysis

**Figure 1:**
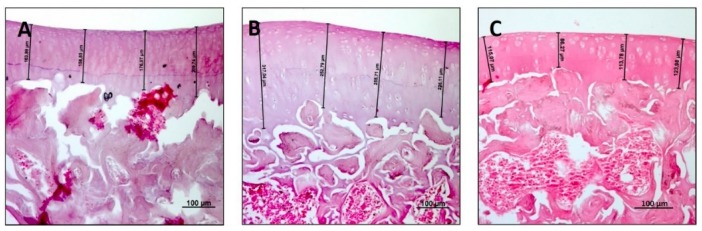
Histological evaluation and histomorphometric analysis of articular cartilage by hematoxylin and eosin (H&E) staining. (**A**) R group (control). In the superficial zone of the articular cartilage, chondrocytes were flat and small; in the middle and deep zone, cells were organised in columns; the tidemark was very strong and evident; (**B**) R-DS group. General tissue preservation was observed in the superficial, middle and deep zones; the tidemark was a little bit less evident, and the deeper zone above the tidemark was very thick; (**C**) R-DR group. Articular cartilage did not show evident alterations; in middle and deep zones the chondrocytes were poorly organised in columns, and the tidemark was not always perceptible. (**A**–**C**) Objective lens, 10; scale bars: 100 μm. These are the most representative images of articular cartilage from rats of each group (R, R-DS and R-DR).

The H&E staining was used to study the general morphology of the tibial articular cartilage in all groups in order to detect eventual alterations and to make a histomorphometric evaluation on the thickness of the articular cartilage. In the R group (control), articular cartilage showed a normal cytoarchitecture. In the superficial zone, cells were flat and small; in the middle and deep zone, chondrocytes were organised in columns; the tidemark was very strong and evident; thickness of articular cartilage in the R group measured 235.7 ± 12.20 μm (Figure 1A). In the articular cartilage of the R-DS group, a general tissue preservation was observed in the superficial, middle and deep zones even if the tidemark was a little bit less evident and the deeper zone above the tidemark was very thick, thus determining a greater overall thickness of the articular cartilage in the R-DS group, measuring 301.6 ± 19.25 μm (Figure 1B). In the R-DR group, though the articular cartilage did not show evident alterations, in its middle and deep zones the chondrocytes were poorly organised in columns, and the tidemark was not always perceptible; moreover, articular cartilage in the R-DR group showed an apparent reduction of thickness, measuring 130.00 ± 13.19 μm (Figure 1C). In the statistical analysis, it emerged that the differences in articular cartilage thickness among groups were statistically significant. In the R-DS group the thickness was greater than both the R group (*p* = 0.0071) and the R-DR group (*p* < 0.0001). The R-DR group had a thickness of articular cartilage lower even if compared to the R group (*p* < 0.0001) (Table 2).

**Figure 2:**
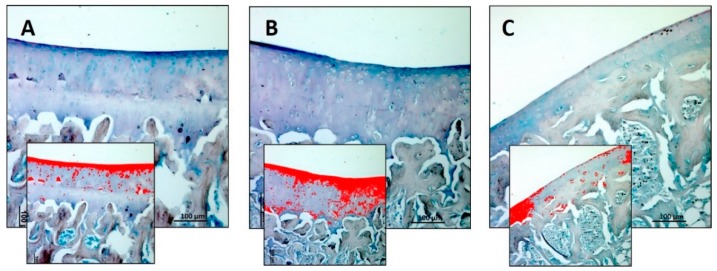
Histochemical evaluation of the mucosubstance content glycosaminoglycan (GAGs) in the articular cartilage by Alcian Blue staining through computerised densitometric measurements and image analysis. (**A**) R group; (**B**) R-DS group; (**C**) R-DR group. In inserts are the image analyses by the software in which the red colour represents Alcian Blue staining, showing a decreased staining in the R-DR group and increased staining in the R-DS group. For details, see the text. (**A**–**C**) Objective lens, 10; scale bars: 100 μm. These are the most representative images of articular cartilage from rats of each group (R, R-DS and R-DR).

The Alcian Blue staining was used to detect and quantify the mucosubstance content (GAGs) in the articular cartilage of all groups. Experimental data obtained using an image analysis software which quantifies the level of Alcian Blue staining expressed as densitometric count (pixel^2^)/μm^2^ of articular cartilage, showed a decreased staining in the R-DR group and increased staining in the R-DS group (Figure 2A–C). In statistical analysis, the difference between R-DS (3.009 ± 0.44) compared to the R group (1.940 ± 0.34) was statistically significant (*p* = 0.0175); the same for the comparison between R-DS compared to the R-DR group (0.980 ± 0.35) (*p* < 0.0001). The R-DR group had a lower value also when compared to the R group (*p* = 0.0359) (Table 3).

**Figure 3:**
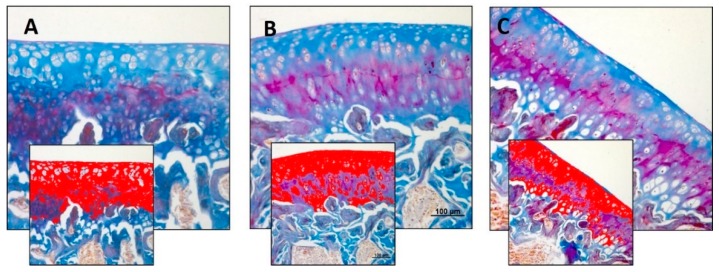
Histochemical evaluation of the fibrous component (collagen fibres) in the articular cartilage by Mallory’s trichrome staining through computerised densitometric measurements and image analysis. (**A**) R group; (**B**) R-DS group; (**C**) R-DR group. In inserts are the image analyses by the software in which the red colour represents blue staining (collagen fibres), showing a decreased staining in the R-DR group. For details, see the text. (**A**–**C**) Objective lens, 10; scale bars: 100 μm. These are the most representative images of articular cartilage from rats of each group (R, R-DS and R-DR).

The Mallory’s trichrome staining was used to highlight the fibrous component (collagen fibres, in blue) of articular cartilage in the experimental groups. Experimental data obtained using an image analysis software which quantifies the level of blue staining, expressed as densitometric count (pixel^2^)/μm^2^ of articular cartilage, showed a decreased staining in the R-DR group when compared to both the R group and R-DS one, and none significant difference between the R-DS group and R one (Figure 3A–C). In statistical analysis, the difference between R-DS (4.834 ± 0.74) compared to the R group (4.246 ± 0.77) was not statistically significant (*p* = ns); not the same for the comparison R-DS compared to the R-DR group (2.925 ± 0.31) (*p* = 0.0003). The R-DR group had a lower value also when compared to the R group (*p* = 0.0120) (Table 4).

### 3.2. Immunohistochemistry (IHC)

Immunohistochemical staining with statistical analysis was used to verify the greater or lesser expression of structural molecules, such as Col II and X, and functional molecules, such as lubricin and VDR. Data were obtained using an image analysis software which quantifies the level of brown staining, expressed as densitometric count (pixel^2^)/μm^2^ of articular cartilage.

**Figure 4:**
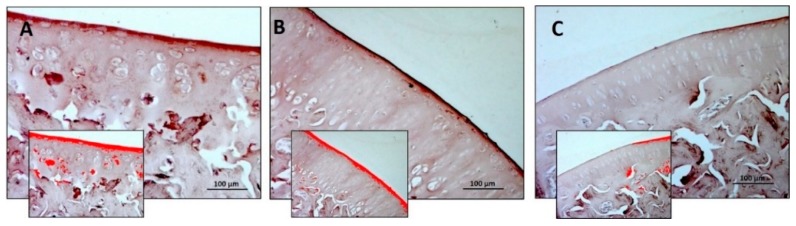
Collagen II (Col II) immunohistochemical evaluation in the articular cartilage through computerised densitometric measurements and image analysis. (**A**) R group; (**B**) R-DS group; (**C**) R-DR group. In inserts are the image analyses by the software in which the red colour represents brown staining (immune complexes labelled with chromogen). For details, see the text. (**A**–**C**) Objective lens, 10; scale bars: 100 μm. These are the most representative images of articular cartilage from rats of each group (R, R-DS and R-DR).

In Col II immunostaining, it was observed that a significant decrease was only in the R-DR group if compared to other experimental groups (R and R-DS groups) (Figure 4A–C). In statistical analysis, the difference between R-DS (0.889 ± 0.15) compared to the R group (0.793 ± 0.20) was not statistically significant (*p* = ns); the difference between R-DS compared to the R-DR group (0.247 ± 0.09) was statistically significant (*p* = 0.0016); also, the difference between R-DR compared to the R group was statistically significant (*p* = 0.0076) (Table 5).

**Figure 5:**
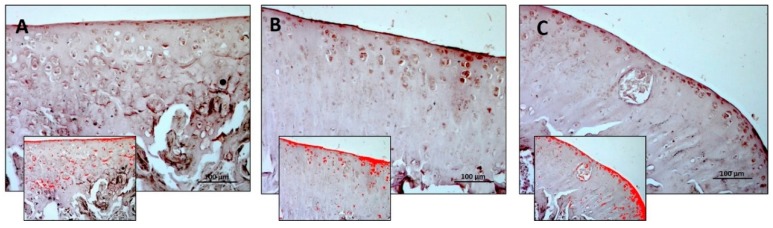
Collagen X (Col X) immunohistochemical evaluation in the articular cartilage through computerised densitometric measurements and image analysis. (**A**) R group; (**B**) R-DS group; (**C**) R-DR group. In inserts are the image analyses by the software in which the red colour represents brown staining (immune complexes labelled with chromogen). For details, see the text. (**A**–**C**) Objective lens, 10; scale bars: 100 μm. These are the most representative images of articular cartilage from rats of each group (R, R-DS and R-DR).

In Col X immunostaining, a higher immunoexpression was evidenced in the R-DR group compared to both R and R-DS groups (Figure 5A–C). In statistical analysis, the difference between R-DR (0.517 ± 0.03) compared to the R group (0.236 ± 0.04) was statistically significant (*p* < 0.0001); also the difference between R-DR compared to the R-DS group (0.300 ± 0.06) was significant (*p* < 0.0001); instead, the difference between R-DS compared to the R group was not statistically significant (*p* = ns) (Table 6).

**Figure 6:**
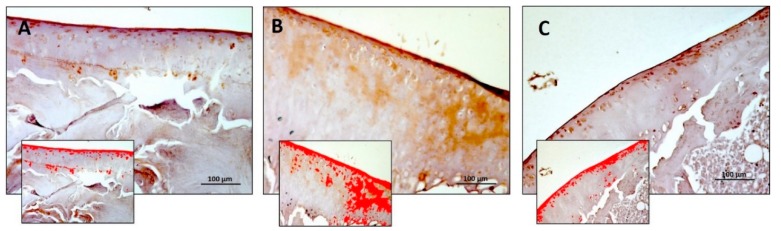
Lubricin immunohistochemical evaluation in the articular cartilage through computerised densitometric measurements and image analysis. (**A**) R group; (**B**) R-DS group; (**C**) R-DR group. In inserts are the image analyses by the software in which the red colour represents brown staining (immune complexes labelled with chromogen). For details, see the text. (**A**–**C**) Objective lens, 10; scale bars: 100 μm. These are the most representative images of articular cartilage from rats of each group (R, R-DS and R-DR).

In lubricin immunostaining, the R-DS group showed higher immunostaining in comparison to both R and R-DR groups (Figure 6A–C). In statistical analysis, the difference between R-DS (2.427 ± 0.35) compared to the R group (1.283 ± 0.34) was statistically significant (*p* = 0.001); the same for comparison between R-DS compared to the R-DR group (1.240 ± 0.50) (*p* = 0.0008); on the contrary, the difference between R-DR compared to the R group was not statistically significant (*p* = ns) (Table 7).

**Figure 7:**
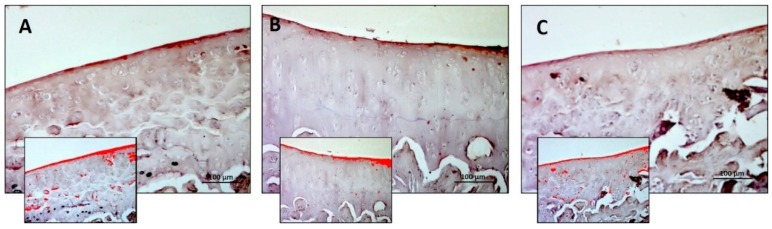
Vitamin D receptor (VDR) immunohistochemical evaluation in the articular cartilage through computerised densitometric measurements and image analysis. (**A**) R group; (**B**) R-DS group. (**C**) R-DR group. In inserts are the image analyses by the software in which the red colour represents brown staining (immune complexes labelled with chromogen). For details, see the text. (**A**–**C**) Objective lens, 10; scale bars: 100 μm. These are the most representative images of articular cartilage from rats of each group (R, R-DS and R-DR).

In VDR immunostaining, no significant differences were shown among experimental groups (R, R-DS and R-DR) (Figure 7A–C). In statistical analysis, the difference between R-DS (0.306 ± 0.05) compared to the R group (0.425 ± 0.08), R-DS compared to the R-DR group (0.347 ± 0.04) and R-DR compared to the R group was always not statistically significant (*p* = ns) (Table 8).

## 4. Discussion

In the present study, the effects of exogenous Vit D supplementation on tibial articular cartilage, in a young healthy sedentary rat model, has been assessed. The results of this study demonstrate a favorable impact of exogenous Vit D supplementation (R-DS group) on the tibial articular cartilage thickness and structure. The impact of the Vit D supplementation has been further highlighted by the opposite results observed in a Vit D restricted diet group (R-DR), where the rats showed a much thinner tibial articular cartilage, supporting the data from literature [24]. An increased ECM and proteoglycan production in the R-DS group, supported both by histochemical and immunohistochemical analysis, determined a greater thickness development of the articular cartilage and a better definition of three cartilage characteristic zones (superficial, middle and deep ones), which might suggest better mechanical properties of the latter. These findings are in accordance with the work by Pascual–Garrido and coauthors which analyzed femoral structure in rats subjected to a modified diet and UV light restriction which provoked Vit D deficiency. Their results highlighted a significant loss of proteoglycan staining in the Vit D-deficient group but no other major histologic changes in cartilage and in the subchondral bone, concluding that low levels of Vit D have a deleterious effect on the articular cartilage [11]. Indeed, it is well known that the mechanical properties of the articular cartilage depend on the composition of the tissue, mainly collagen and proteoglycans, and their ultrastructural and molecular organization. Since proteoglycans are rich in fixed negative charges, the articular cartilage exhibits a significant osmotic pressure, which contributes to the total swelling pressure together with charge-to-charge repulsive forces exerted by the closely spaced negative charge groups along the proteoglycan molecules [25]. The more intense Alcian Blue staining in the R-DS group, when compared to the R-DR group, testifies that the amorphous substance of the ECM is particularly rich in GAGs, responsible for the physicochemical properties of the cartilage, responding better to the mechanical stress to which it is typically subjected. Mallory’s trichrome staining was also evaluated to highlight the fibrous component (collagen fibers) of articular cartilage in the experimental groups. No significant differences in blue staining (which identifies the collagen fibers) were evidenced between the R-DS group and the control group (R), whereas in the R-DR group a weaker blue staining could be seen, suggesting a depletion in collagen fiber production resulting from an insufficient intake of Vit D with diet, as expected.

Furthermore, through immunohistochemical staining, the expression of some specific chondrocyte markers, such as Col II and lubricin, and a hypertrophic marker such as Col X, have been evaluated. The results of immunohistochemistry confirmed the results of Mallory’s trichrome staining. Indeed, there was no higher expression of Col II in the R-DS group when compared to the control group R. Instead, the R-DR group had a lower Col II immunostaining both in comparison with the R group and the R-DS group. Moreover, the R-DS group showed increased immunostaining of lubricin, whose role is to enhance joint function, by greater tissue lubrication [26,27]. The R-DR group showed a decreased lubricin immunostaining, even if the difference was not significant when compared to the control group (R). It was also observed that lubricin, in the R-DS group, was localised in both ECM and cytoplasm of chondrocytes, while it was exclusively expressed in the chondrocyte cytoplasm of the R and R-DR groups. Concerning Col X, a higher immunoexpression was evidenced in the R-DR group compared to both R and R-DS groups, further confirming that the Vit D restriction in the diet leads to cartilage hypertrophy and may trigger the OA onset [28].

Finally, the expression of VDR was evaluated by immunohistochemistry to assess if the increased expression of lubricin in the articular cartilage might be directly connected to the increased VDR expression and activation, following the link with Vit D to the latter. The results were not as expected. No significant differences in the VDR immunostaining were found among the groups (R, R-DS and R-DR). Although the results concerning VDR were not as expected, this does not exclude that there could be a more significant number of VDRs activated by the higher local presence of Vit D, which may exert its effect on the articular cartilage tissue, further highlighted by the higher expression of lubricin, Col II and ECM proteoglycans.

There are several studies suggesting that the Vit D deficiency is associated with the decreased articular cartilage thickness as well as with the increased risk of cartilage degeneration and, usually, with the OA onset. However, even if the symptoms of Vit D deficit are largely evidenced in literature referred to cartilage diseases, the novelty of this present study is that it has been assessed some new parameters/biomarkers (such as Col II and X, lubricin and VDR expression) not evidenced in literature in the same experimental conditions. As expected, the results strengthen the concept of the detrimental effect of Vit D deficiency on cartilage tissue, especially during the developmental period in young age. On the contrary, there is a poor evidence in literature concerning the effects of the Vit D-enriched diet on the articular cartilage, particularly, in healthy young sedentary subjects. For example, the beneficial effects of Vit D have been recently demonstrated in an interesting study by Rai et al., where the Vit D supplementation attenuated the inflammation and fatty infiltration in joint tissues, in the microswine model subjected to high cholesterol diet, suggesting its importance in protecting the architecture of the articular cartilage [29]. In the present study, as described in the results, a novelty is that the Vit D-supplemented diet may support the healthy articular cartilage and its tribology in young age and in healthy animals.

Overall, in light of these findings, authors imply that rats fed with a diet supplemented with Vit D have thicker tibial cartilage when compared to the control and, especially, to those with Vit D restriction, which tibial cartilage appear much thinner and which molecular structure results altered. Moreover, the higher expression of the functional molecule as lubricin induced by the Vit D-supplemented diet is a relevant scientific data supporting the beneficial effect on morphology/physiology of the articular cartilage. Although the results of the present study are encouraging in relation to the role of the Vit D supplementation in the diet, meta-analysis of randomized controlled trials showed moderate effect deriving from Vit D supplementation on pain, functionality and disease progression among patients with knee OA [30,31]. The results of these studies indicate that Vit D supplementation may not have a clinically significant effect on patients with a definite diagnosis of knee OA. The clinical relevance of the present study could be recognized considering the supplementation of Vit D as a non-pharmacological treatment in early osteoarthritic patients with a non-established diagnosis, since it has been shown that Vit D has different effects on cartilage morphology and molecular composition in healthy rats. Authors suggest that further studies are warranted to assess the clinical relevance of this change in cartilage thickness consequent to different exogenous Vit D provision, especially during the developmental period in young individuals and to further explore whether Vit D supplementation may allay clinical symptoms in the course of OA.

## 5. Conclusions

The results obtained in the present study show some preliminary data on the influence of introducing Vit D supplementation in the daily diet in standard health conditions. The results of this study support the idea of functional foods and their positive effects on tissue homeostasis, especially on the articular cartilage during its developmental period in young age. Today, a healthy diet is a fundamental topic for the physical well-being of people. Authors believe and hope, therefore, that this study may contribute to deepen this item and to constitute a preliminary data for further studies necessary to define the appropriate quantities of Vit D to be supplemented in the diet to be used as a preventive tool for the articular cartilage diseases. So, in the future, we would like to consider the effects of dietary Vit D supplementation on animals that have undergone a previous Vit D restriction, to verify whether the exogenous Vit D supplementation may reverse or attenuate the cartilage degeneration.

## 6. Limitations of the Study

There are some limitations of the present study. One of them is represented by the lack of Vit D dosages in the biological fluids of animals, such as blood and/or synovial fluid, for a stronger proof of the presence of Vit D in its active form (1,25(OH)_2_D_3_). Moreover, only male rats were used; therefore, the results cannot be extrapolated to females. Lastly, sample size is quite small according to the 3 Rs rule (Replacement, Reduction, Refinement) for more ethical use of experimental animals, surely recruiting more animals might sharpen the differences between the other groups as well. Furthermore, the effects on the number/density of the chondrocytes have not been evaluated. Further investigations will be required keeping in mind these limitations to better investigate the mechanism of action of Vit D in the articular cartilage in young age.

## Figures and Tables

**Table 1:** The composition of the experimental diets.

Compound	R	R-DS	R-DR
Water (% *w*/*w*)	10.69	10.69	10.69
Protein (% m/m)	22.90	22.90	22.90
Fat (% m/m)	3.54	3.54	3.54
Fiber (% m/m)	3.63	3.63	3.63
Ash (% m/m)	7.55	7.55	7.55
FNE (% m/m)	51.44	51.44	51.44
Carbohydrates (% m/m)	55.07	55.07	55.07
M.E. (kcal/kg)	2757	2757	2757
Vitamin A (IU/kg)	16,000	16,000	16,000
Vitamin D_3_ (IU/kg)	1400	4000	0
Vitamin B1 (mg/kg)	19.1	19.1	19.1
Vitamin B2 (mg/kg)	19.1	19.1	19.1
Vitamin B6 (mg/kg)	10.9	10.9	10.9
Vitamin B12 (mg/kg)	0.03	0.03	0.03
Vitamin E (α-tocopherol) (mg/kg)	68.5	68.5	68.5
Vitamin PP (mg/kg)	95.2	95.2	95.2
Iron (mg/kg)	599	599	599
Calcium (mg/kg)	10,575	10,575	10,575
Magnesium (mg/kg)	2485	2485	2485
Phosphorus (mg/kg)	9619	9619	9619
Potassium (mg/kg)	9782	9782	9782
Zinc (mg/kg)	106.9	106.9	106.9
Sodium (mg/kg)	3033	3033	3033

M.E.: Metabolizable Energy; R: Regular diet; R-DS: Regular diet with vitamin D supplementation; R-DR: Regular diet with vitamin D restriction.

**Table 2:** THICKNESS—Articular cartilage.

	R (Mean ± SD)	R-DS (Mean ± SD)	R-DR (Mean ± SD)	*p*-Value
Thickness (µm)	235.7 ± 12.20	301.6 ± 19.25	130.00 ± 13.19	R vs. R-DS: 0.0071 R vs. R-DR: < 0.0001 R-DS vs. R-DR: < 0.0001

**Table 3:** ALCIAN BLUE Staining—Articular cartilage.

	R (Mean ± SD)	R-DS (Mean ± SD)	R-DR (Mean ± SD)	*p*-Value
Densitometric count (pixel^2^)/μm^2^	1.940 ± 0.34	3.009 ± 0.44	0.980 ± 0.35	R vs. R-DS: 0.0175 R vs. R-DR: 0.0359 R-DS vs. R-DR: < 0.0001

**Table 4:** MALLORY’S Trichrome Staining—Articular cartilage.

	R (Mean ± SD)	R-DS (Mean ± SD)	R-DR (Mean ± SD)	*p*-Value
Densitometric count (pixel^2^)/μm^2^	4.246 ± 0.77	4.834 ± 0.74	2.925 ± 0.31	R vs. R-DS: ns R vs. R-DR: 0.0120 R-DS vs. R-DR: 0.0003

**Table 5:** COL II Immunostaining—Articular cartilage.

	R (Mean ± SD)	R-DS (Mean ± SD)	R-DR (Mean ± SD)	*p*-Value
Densitometric count (pixel^2^)/μm^2^	0.793 ± 0.20	0.889 ± 0.15	0.247 ± 0.09	R vs. R-DS: ns R vs. R-DR: 0.0076 R-DS vs. R-DR: 0.0016

**Table 6:** COL X Immunostaining—Articular cartilage.

	R (Mean ± SD)	R-DS (Mean ± SD)	R-DR (Mean ± SD)	*p*-Value
Densitometric count (pixel^2^)/μm^2^	0.236 ± 0.04	0.300 ± 0.06	0.517 ± 0.03	R vs. R-DS: ns R vs. R-DR: < 0.0001 R-DS vs. R-DR: < 0.0001

**Table 7:** LUBRICIN Immunostaining—Articular cartilage.

	R (Mean ± SD)	R-DS (Mean ± SD)	R-DR (Mean ± SD)	*P*-Value
Densitometric count (pixel^2^)/μm^2^	1.283 ± 0.34	2.427 ± 0.35	1.240 ± 0.50	R vs. R-DS: 0.001 R vs. R-DR: ns R-DS vs. R-DR: 0.0008

**Table 8:** VDR Immunostaining—Articular cartilage.

	R (Mean ± SD)	R-DS (Mean ± SD)	R-DR (Mean ± SD)	*p*-Value
Densitometric count (pixel^2^)/μm^2^	0.425 ± 0.08	0.306 ± 0.05	0.347 ± 0.04	R vs. R-DS: ns R vs. R-DR: ns R-DS vs. R-DR: ns
